# From Meows to Moos: Recruiting Teens to Food Animal Veterinary Medicine Through Experiential Camps

**DOI:** 10.3390/vetsci13020137

**Published:** 2026-01-30

**Authors:** Marissa Hall, Jacqueline M. Nolting

**Affiliations:** Department of Veterinary Medicine, College of Veterinary Medicine, The Ohio State University, Columbus, OH 43210, USA; hall.3043@osu.edu

**Keywords:** camps, high school, recruitment, food supply veterinary medicine, recruiting high school students

## Abstract

Food supply veterinarians, those who service the dairy, swine, poultry, small ruminant, and beef cattle industries, benefit society by protecting animal and public health and ensuring a safe, wholesome food supply. However, there are not enough entering the workforce to meet current and future demands. Non-formal learning environments can be used as a recruitment tool to provide participants with positive interactions and hands-on experiences. To build awareness of food supply veterinary medicine (FSVM) in youth, we developed an immersion program designed to provide high school students with hands-on experiences with food animal species. Day camps were held during the summers of 2022 and 2023, each coordinated with multiple partners at different locations in central Ohio. Year One camp utilized registration and post-test surveys and Year Two utilized matching pre- and post-test for analysis. Over the two programs, 110 participants engaged in hands-on experiences, including: outbreak investigations, measuring clinical parameters, performing diagnostics, and basic veterinarian procedures. Pre- and post-test evaluations were performed to measure changes in participants’ attitudes and perceptions, and a McNemars test was used to evaluate Year Two data. In Year One, we saw positive shifts in those interested in FSVM careers. In Year Two, we saw positive shifts in knowledge of FVSM careers, with biosecurity knowledge increasing. Outreach activities like day camps can be replicated in other locations to increase interest in FSVM careers.

## Simple Summary

It is important that we have a safe and wholesome food supply to feed our growing population. To provide healthy animals for the food supply, we need food supply veterinarians to ensure the health and welfare of the animals and safety of food products, as veterinarians play crucial roles in every aspect of animal production. However, in recent years, fewer veterinarian students are choosing careers in food supply medicine, resulting in workforce shortages. To fill this gap, we sought to build awareness of career opportunities in food supply veterinarian medicine with high school students by hosting food animal focused veterinary camps. Youth in urban areas were recruited for participation in the camps, as they may not have experience with food animals or be familiar with careers in food supply veterinary medicine. By exposing these participants to large animals, we hypothesize they will be more likely to develop an interest in this career field. Upon completion of camp, participants reported increased knowledge in opportunities in this career field, the importance of the career field, and biosecurity knowledge.

## 1. Introduction

Food supply veterinarians have an important role in protecting and promoting public health, serving as leaders at the interface of human, animal, and environmental health. They also help in ensuring animal welfare, food safety, pharmacovigilance, and many other important roles to keep the agriculture industry safe. Their role is indispensable in the mitigation of emerging and endemic zoonotic diseases, which pose a persistent threat to global health security. Current data indicates that approximately 61% of all human infectious diseases and up to 75% of emerging infectious diseases originated in animal populations [1]. This biological reality necessitates a “One Health” framework, which is a collaborative, multidisciplinary approach that recognizes that the health of people is closely connected to the health of animals and our shared environment. Efforts to control infectious cycles that focus exclusively on human clinical outcomes while neglecting the animal reservoir are reactionary and fail to achieve long-term prevention [2].

Simultaneously, the veterinary profession is tasked with animal production that must increase to meet the nutritional needs of a growing population while ensuring the health and safety of each consumer, ensuring global food security. As the global population is projected to reach nearly 10 billion by 2050, food production systems must scale to meet nutritional demands. While animal-based proteins remain a vital component of the human diet, their production carries significant risks regarding foodborne pathogens and chemical contaminants. Ensuring that livestock systems remain productive yet safe requires the diligence of food supply veterinary medicine (FSVM) professionals to oversee herd health and ensure strict compliance with slaughterhouse regulations [3]. Central to this is the Veterinary Client Patient Relationship (VCPR), which serves as the legal and ethical foundation for all veterinary interventions on the farm.

A critical facet of the FSVM practitioner’s role is the promotion of biosecurity and the stewardship of pharmaceutical resources. Veterinarians are at the forefront of designing farm-specific biosecurity protocols that minimize the transmission of pathogens [4]. Furthermore, the management of drug residues is a paramount public health concern, as violative residues in meat, milk, or eggs can trigger allergic reactions or contribute to chronic health issues in consumer populations. Veterinarians must not only prescribe medications for therapy and prophylaxis but also serve as educators, ensuring that producers strictly adhere to withdrawal periods and make sure products have the intended effects [5,6].

In the United States, the regulatory landscape shifted significantly in 2017 with the full implementation of the FDA’s Veterinary Feed Directive (VFD). This policy ended the use of medically important antimicrobials for growth promotion and mandated that any such drugs administered via feed or water be so administered under the direct supervision of a licensed veterinarian within a valid VCPR [7,8]. This shift was a direct response to the global crisis of antimicrobial resistance (AMR). AMR occurs when bacteria evolve to survive the effects of an antibiotic, often accelerated by improper or extra-label drug use [9]. By ensuring that antimicrobials are used judiciously and only when clinically indicated, FSVM practitioners act as the final line of defense against the development of superbugs that threaten both veterinary and human medicine [6].

Despite their importance, the United States faces a chronic shortage of food supply veterinarians, particularly in rural and underserved agricultural regions. There has been a 90% decrease in this field since World War II [10]. Addressing this shortage requires a longitudinal “pipeline” strategy that engages potential practitioners at every stage of their development. Within the professional veterinary curriculum, recruitment and retention are bolstered by hands-on clinical exposure to large animal medicine, paid summer internships, and structured mentorship programs [11,12]. Post-graduation, federal and state-level incentives, such as the Veterinary Medicine Loan Repayment Program (VMLRP), seek to alleviate the significant debt-to-income ratio that often deters new graduates from entering food animal practice [13].

However, internal professional incentives are insufficient if the pool of incoming students remains narrow. To truly expand the workforce, outreach must begin in secondary education. Non-formal educational activities, learning experiences that occur outside the traditional, credit-bearing classroom, offer a unique opportunity to pique student interest in specialized STEM fields. Academic camps, in particular, have been shown to improve student self-efficacy and clarify career trajectories by providing immersive, low-stakes environments for exploration [14].

Non-formal learning environments allow for a pedagogical shift from rote memorization to experiential inquiry. By removing the pressure of high-stakes testing, these environments enable students to focus on the application of scientific concepts in real-world scenarios [15]. For these programs to be effective, they must prioritize diversity, equity, and inclusion. Utilizing a diverse cohort of mentors and speakers representing various races, genders, and socioeconomic backgrounds validates the career aspirations of underrepresented students and dismantles the image of agriculture as an inaccessible or homogenous field [16,17,18].

Many students do not want to go into post-secondary agriculture education programs due to scheduling, lack of guidance, and the image of agriculture [19]. To further expand the pool of students interested in FSVM careers, recruitment and outreach should also target students in urban areas, who may not have been previously exposed to livestock and agriculture. A workshop that targeted underrepresented inner-city high school students was able to increase knowledge and understanding of agriculture, including career paths [20]. Another program, which educated people from urban areas about agriculture, evaluated participants long term and found that the program did alter their career aspirations toward agriculture careers in a positive way [21]. In addition, Ester et al. studied graduates from specialized urban agriculture programs located in Midwestern states to evaluate their career and educational pursuits after graduation. These schools are found in major cities across the Midwest and emphasize urban agriculture to attract more urban, minority, and non-minority students into agriculture education [22]. They found that only 8% of students who graduated pursued degrees in agriculture and fewer held a master’s degree in an agricultural subject [22].

Currently, there is a significant gap in the literature regarding the efficacy of veterinary-specific camps for high school students. Most existing research focuses on younger age groups or general STEM camps, with only one major peer-reviewed study evaluating a veterinary camp for 8th graders through qualitative essay analysis [23]. To address this gap, we developed “Be a Vet for a Day,” a cost-free immersion program specifically targeting 11th and 12th-grade students from urban environments. We hypothesize that targeted exposure to food animal clinical skills and FSVM career pathways will significantly increase the likelihood of these students to take an interest in FSVM as a possible career aspiration. To gauge participants’ thoughts toward FSVM careers, 4-point Likert scales were be utilized. This study reports on the outcomes of two camp iterations (2022 and 2023) held in partnership with the Ohio State Fair and the OSU College of Food, Agricultural, and Environmental Sciences (CFAES). By engaging these students at a pivotal point in their academic decision-making, we seek to cultivate a more diverse, robust, and qualified future food supply veterinary workforce.

## 2. Materials and Methods

### 2.1. Study Design and Camp Recruitment

The “Be a Vet for a Day” camp was developed through a multi-institutional partnership including The Ohio State University (OSU) Extension, the Ohio Veterinary Medical Association (OVMA), the Ohio State Fair (OSF), and the OSU Colleges of Veterinary Medicine (CVM) and Food, Agricultural, and Environmental Sciences (CFAES).

The program targeted 11th and 12th-grade students from urban areas to promote interest in food supply veterinary medicine (FSVM) and livestock species. Other age groups were able to sign up, although flyers and other materials were advertising for 11th and 12th-gradets. Urban areas were defined as an area with more than 50,000 people in the population and in a city center. Since many individuals choose careers based on background and personal experiences, such as the type of animals they were exposed to growing up, we sought to give participants positive experiences with livestock species rather than companion animals they may already know [24]. Recruitment for Year 1 (2022) utilized QR-coded flyers distributed via county extension offices and social media. In Year 2 (2023), recruitment focused on urban Boys and Girls Clubs and local health departments. As there was no identifiable information taken from participants, institutional review board support was granted for this project. Informed consent was given when participants took the pre- and post-test surveys. All protocols were approved by the Institutional Review Board (2022E1030 and 2023E0816).

### 2.2. Curriculum Design and Development

A “backwards design” methodology was employed: identifying FSVM career paths, establishing learning objectives, creating pre- and post-test assessments, and developing experiential learning activities. Year 1 sessions were held at the OSF (half-day), while Year 2 sessions were held at the OSU Beef and Equine Center and CVM teaching hospitals (full-day). Career areas included in camp sessions included; general practice, regulatory veterinarians (e.g., Ohio Department of Agriculture field veterinarians), extension veterinarians, veterinary technicians, and fair veterinarians.

The year one camp was designed to be a half-day event with participants receiving free entrance to the OSF. During this time, participants were divided into groups and rotated through four interactive sessions, 45 min each, with camp ending mid-day. This event had a lead presenter for each of the four groups, and each leader brought additional staff depending on the intensity of the session.

The programming in Year Two was altered to accommodate additional activities. The agenda began with presentations from a FSVM veterinarian and a small ruminant extension specialist to discuss their experience in FSVM and what careers are needed in the future. The remainder of the event included three 45 min rotating sessions in the morning that consisted of cattle herd health, equine health, and biosecurity with a large animal focus, and afternoon tours of The Ohio State University CVM teaching hospitals. For the three rotating sessions in the morning, there was a lead and an extra staff member in the session to help to conduct activities. Participants were transported from the beef and equine center to the teaching hospital upon completion of release forms by parent or guardians of minors. Participants were split into groups and were led through the small animal hospital, the large animal hospital, and the clinical skills laboratory by a staff member from each section of the hospital. One extra staff member went with each of these groups.

Admission recruiters from The Ohio State University CVM and CFAES had displays at the event to engage with participants. Parents were also encouraged to interact with the recruiters while participants were in the session to get more information on application processes and learning opportunities through the programs. CFAES also provided pamphlets for participants to take about their undergraduate program at The Ohio State University for those who were interested. Each participant received a welcome bag that had free items including promotional gear, camp t-shirts, and printed information about FSVM and Ohio State’s CVM. A notes page was also provided in the bag, so participants could record what they had learned at the camp.

### 2.3. Camp Sessions Year 1

#### 2.3.1. Session 1

This session, led by the Assistant State Veterinarian from the Ohio Department of Agriculture, gave participants insight on how government agencies conduct investigations of on-site outbreaks of reportable diseases. In this session, participants donned and doffed personal protective equipment (PPE) and simulated a disease outbreak response. For the PPE portion, each participant received a Tyvek suit, gloves, boot covers, and hairnets. Participants were first given oral instructions and were shown how to don PPE. Participants then swabbed and tested a stuffed pig to simulate outbreak response. Participants then learned proper doffing procedures and discussed why animal officials don and doff PPE and how they handle a real outbreak investigation.

#### 2.3.2. Session 2

Session two was led by veterinary technicians from The Ohio State University Veterinary Medical Center, who utilized clinical simulations typically reserved for veterinary students. The stations focused on equine medicine—including nasogastric intubation, limb bandaging, venipuncture, and suturing—though the underlying principles of these techniques are broadly applicable to population health across various large animal species. These skills were practiced on models that were provided and designed by The Ohio State University clinical skills labs in the veterinary medicine college.

To maximize engagement, participants were divided into small cohorts for a structured rotation through each station. These simulations provided a unique opportunity for hands-on learning that is often inaccessible to pre-veterinary students. Furthermore, using high-fidelity models addressed the ethical and safety concerns associated with performing invasive procedures on live, healthy animals. By practicing on the same equipment used in the professional curriculum, participants gained a realistic preview of veterinary training, thereby increasing their clinical confidence and demystifying the technical skills required for a career in large animal medicine.

#### 2.3.3. Session 3

The third session, facilitated by a large animal clinician from The Ohio State University, integrated traditional clinical diagnostics with modern veterinary technology. Participants were introduced to the role of innovation in the field through a demonstration of handheld ultrasonography, where they applied the technology to evaluate ovine anatomy in real-time. This was followed by a comprehensive tutorial on ruminant physical examinations, emphasizing the assessment of vital parameters including heart rate, respiratory rate, and rumination frequency.

Under veterinary supervision, students entered the pens to perform these examinations using stethoscopes, gaining direct experience in handling live animals. When time permitted, the curriculum was expanded to include hoof trimming, a fundamental aspect of small ruminant husbandry. For many participants, particularly those from urban backgrounds, this represented their first tactile interaction with livestock. By performing these daily veterinary tasks, students not only developed foundational clinical skills but also improved their confidence in large animal handling, which is a critical prerequisite for pursuing food animal veterinary medicine.

#### 2.3.4. Session 4

The final session was led by a large animal veterinarian and doctoral researcher focusing on bovine metabolic monitoring. Participants were guided through the clinical application of ketone testing in dairy cattle, an essential herd management tool used to diagnose ketosis. The session highlighted the importance of early detection in maintaining nutritional balance and optimizing lactation performance.

Due to the invasive nature of the procedure, specifically a coccygeal (tail vein) blood draw, and the use of a privately owned exhibition animal, participants engaged in observational learning rather than direct practice. The presenter demonstrated the technique and the use of rapid, commercialized diagnostic tests to show how point-of-care technology supports dairy herd health. This demonstration illustrated the practical integration of diagnostic medicine and producer consultation, underscoring the veterinarian’s role in enhancing farm productivity through routine metabolic surveillance.

### 2.4. Sessions Year 2

#### 2.4.1. Session 1

Led by an Associate Professor and a Research Assistant from The Ohio State University, this session provided a comprehensive overview of the beef cattle production cycle. Participants toured three distinct units to observe various management phases: a steer finishing group, a cow-calf pair, and feedlot cattle nearing slaughter weight. The curriculum integrated physiological concepts such as the examination of preserved cross-sectional organs (heart, brain, and kidney) with behavioral principles like flight zones and herd health basics.

Discussion at the cow-calf and feedlot units covered life-stage-specific environmental requirements, age and weight estimation, identification methods, and common pathologies, including bovine papillomavirus. Although this session was primarily observational due to the production environment, it utilized inquiry-based learning to bridge the gap between the participants’ existing biological knowledge and the complexities of livestock production. For students from non-agricultural backgrounds, this immersive tour offered critical insight into the veterinarian’s role in maintaining the health and welfare of animals throughout the food supply chain.

#### 2.4.2. Session 2

Facilitated by an assistant professor from The Ohio State University, this session focused on the principles of biosecurity and infectious disease mitigation. The curriculum established the role of biosecurity in safeguarding both animal and public health before transitioning to a practical demonstration of sanitation efficacy. Participants used “Glo Germ” fluorescent paint on silicone models to simulate microscopic pathogen contamination. By attempting to scrub the paint clean and inspecting the models under ultraviolet (UV) light, students visualized the disparity between cleaning and disinfection, which is crucial in clinical veterinary practice.

As a culminating activity, participants assembled “biosecurity kits” to take home, which included personal protective equipment (PPE), disinfectants, and sanitation tools such as scrub brushes and boot covers. This hands-on exercise introduced students to the practicalities of field-based veterinary medicine, where practitioners visiting multiple farms daily must adhere to strict biosecurity protocols to prevent the mechanical transmission of pathogens. By exposing participants to these concepts early in their training, the session underscored the veterinarian’s role in proactive herd health and biosecurity stewardship.

#### 2.4.3. Session 3

The third session, facilitated by a practicing equine clinician and a 4-H program specialist, focused on foundational equine husbandry and clinical diagnostics. Participants were divided into cohorts for a dual-track rotation, ensuring individualized instruction in both veterinary and management practices. Under the clinician’s guidance, students performed physical examinations, gaining proficiency in auscultating cardiac and gastrointestinal sounds and recording rectal temperatures. Simultaneously, the program specialist led a practicum on equine handling and hoof health, where students practiced hoof care and discussed appropriate maintenance schedules.

This session emphasized experiential learning, allowing every participant to perform each task directly. By providing this immersive environment, the program helped students overcome barriers to large animal handling and build clinical confidence. Furthermore, the session highlighted the cross-species utility of physical examination techniques, demonstrating how these core competencies serve as the building blocks for comprehensive veterinary care in any large-animal field.

#### 2.4.4. Session 4

Following the morning sessions, participants transitioned to the Veterinary Medical Center (VMC) for structured tours of the small animal hospital, the Galbreath Equine Center, and the clinical skills laboratory. These brief, high-impact rotations provided a comprehensive overview of the tertiary-level care provided at a teaching hospital. In the small animal and equine facilities, participants observed clinical operations and current inpatient treatments, gaining exposure to high-caliber veterinary environments that many had not yet experienced through traditional shadowing.

The rotation concluded in the clinical skills laboratory, where students examined the high-fidelity simulators used for veterinary training. This included a demonstration of the internal development of these models, offering a unique perspective on the intersection of technology and medical education. Collectively, these tours served to demystify the professional curriculum and allowed participants to visualize their potential trajectories as veterinary students within the Ohio State University infrastructure.

### 2.5. Data Collection and Instrumentation

In Year One, an online Qualtrics survey collected demographic data (age, grade, residence, and career interest) at registration. Post-camp paper surveys utilized 4-point Likert scales to assess perceptions of the presenters and overall camp satisfaction. To ensure completion, surveys were exchanged for meal vouchers.

In Year Two, a pre-test/post-test design was implemented using matched-ID paper surveys. To match ID, each participant that came to the camp was given a number upon registration that was written on the participant’s registration packet. When instructed to fill out the post-test, participants were prompted to put the number given to them in the top right corner of the post-test paper. The pre-test collected demographics (age, sex, race, ethnicity) and baseline Likert-scale responses regarding FSVM interest. The post-test was administered during transit from the clinical site, repeating the Likert-scale items and assessing specific session impact.

Likert scales for the 2022 post-test and the 2023 pre- and post-test were newly developed for this camp. All Likert scales were a 4-point scale that allowed participants to strongly agree, agree, disagree, and strongly disagree with each statement. No pilot testing was done for these scales; validation occurred through other researchers’ feedback on the scales before the tests were administered to the participants.

### 2.6. Statistical Analysis

Data were analyzed using STATA IC 15. Descriptive statistics summarized participant demographics. To evaluate changes in knowledge and perception between the pre- and post-tests, McNemar’s tests were utilized for paired categorical data. Any incomplete survey was not used, and five participant surveys were not used in data analysis (*N* = 36). “Strongly agree” and “agree” were combined and “disagree” and “agree” were combined to make dichotomous outcomes to input into the McNemar’s test. Statistical significance was defined as *p* < 0.05.

## 3. Results

### 3.1. Registration Results Year 1

Following the recruitment period (May–June 2022), 112 participants registered for the program, representing 24 Ohio counties (Clark, Delaware, Franklin, Auglaize, Belmont, Fairfield, Highland, Champaign, Clinton, Darke, Fulton, Ashland, Ashtabula, Athens, Clermont, Columbiana, Crawford, Cuyahoga, Greene, Greenup, Guernsey, Hancock, Huron, Lewis) and one county in North Carolina (Cumberland). The geographic distribution was concentrated in central Ohio, with Clark County providing the highest number of registrants (*N* = 8). While 112 students registered, the total responses for specific demographic and attitudinal questions varied (*N* = 109), as the survey instrument did not utilize forced-response settings.

Prior to the intervention, 61.6% (*N* = 69) of registrants indicated a primary interest in large animal medicine. In contrast, none of the participants initially expressed interest in government or regulatory veterinary medicine. A significant portion of the cohort (19.6%, *N* = 22) remained undecided regarding their intended career path. The remaining registrants indicated preferences for small animal medicine (12.5%, *N* = 14) or “other” specialized tracks (6.3%, *N* = 7), including exotic animal medicine, mixed-practice, swine medicine, and veterinary research. Although the program specifically targeted 11th and 12th graders, eligibility was expanded to students entering grades 9 through 12 to ensure a robust sample size. Detailed demographic data for all registrants are summarized in Table 1.

### 3.2. Registration Results Year 2

After the five-day enrollment period, 78 participants registered for the camp. In this survey, personal information such as phone numbers, email address, food restrictions, and T-shirt sizes was collected.

#### 3.2.1. Survey Results Year 1

Of the 112 participants registered, 79 participants came to the camp. Post-survey results were available for 69 participants, for a response rate of 87.3% of those who came to the camp. This camp was intended to target participants from urban areas. In the 2022 camp, a large demographic (60.3%) came from the farm population and 39.8% from non-farm demographics.

For the pre-test registration, participants were asked general demographic questions such as county of residence, grade, and age. Participants were also asked what field of veterinary medicine interested them the most. All participants answered these questions to register for the camp. Participants were asked if after the camp they were aware of opportunities in food animal medicine, would consider a career in food animal medicine, planned to pursue a career in food animal medicine, and if they would share information that they learned at the camp. When asked if participants were aware of opportunities in veterinary medicine, 98% of participants strongly agreed or agreed that they were now aware of these opportunities, with only one participant disagreeing. Many participants (94%) indicated that they strongly agreed or agreed with considering a career in FSVM. When asked if they planned to pursue a career in food animal veterinary medicine, 80% of participants strongly agreed or agreed that they were planning on pursuing a career in food animal medicine. There were twelve participants who disagreed with this statement and two who strongly disagreed. Finally, when asked whether they would share what they learned at this camp, the majority (97%) strongly agreed or agreed that they would share information. In assessing the camps’ impacts on participants’ career choices, we applied a 2 × 2 table, as shown in Table 2. This table uses the registration data in which participants indicated their preferred career path in veterinary medicine. This was compared to the post-survey to detect any change in intended career path after this camp.

Participants were also asked to rate the session leaders on a 4-point Likert scale; 69 participants participated in this rating. For Sessions 1–3, all participants indicated that they strongly agreed or agreed that the sessions were informative. For Session 4, more than half of participants indicated that they strongly agreed or agreed that the session was informative, with three disagreeing that the session was informative and one strongly disagreeing that it was informative.

When participants were asked about their favorite session, 58.2% of respondents stated that Session 2, which was comprised of the veterinary technician team, was their favorite session of the camp. Session 3, the general practice veterinarian measuring clinical parameters on sheep, was the second favorite of participants, then session 1, the Assistant State Veterinarian, and lastly session 4, the general practice veterinarian performing ketone tests.

#### 3.2.2. Survey Results Year 2

Of the 78 participants registered, 41 participants came to the camp. We were able to collect 40 pre-surveys and 41 post-surveys. Demographic data for the Year One and Year Two participants (reported on the pre-surveys) are reported below in Table 3.

Additionally, we asked participants six Likert-like scale questions in the pre- and post- survey to look at participants’ perceptions before and after the camps. Results for these questions can be seen in Table 4.

Two questions were shown to be statistically significant from the pre- and post-test, with a *p*-value less than 0.05 as reported in Table 5. First, Question 3, with students shifting from agree/strongly agree to disagree/strongly disagree when asked if they were planning to pursue a career in food animal veterinary medicine. The second statistically significant response was from question six, in which participants response shifted from disagree/strongly disagree to agree/strongly agree when asked about their understanding of biosecurity on farms.

## 4. Discussion

The “Be a Vet for a Day” program successfully achieved its primary objective of immersing pre-college students in the multifaceted career paths of food supply veterinary medicine (FSVM). By targeting high school juniors and seniors (mean age 16), the program engaged a cohort at a critical developmental juncture for making undergraduate and professional education decisions. While we had some participants outside of these grades, our mean for both years still stayed 16. The participant gender distribution (87–88% female) aligns with current enrollment trends at The Ohio State University College of Veterinary Medicine (86% female) [25]. While these figures reflect the current demographic shift within the profession, they highlight the ongoing need for targeted recruitment strategies to diversify gender representation specifically within the food animal sector.

Significant refinements were made to the evaluative framework between the 2022 and 2023 iterations. In 2022, registration data were utilized as a proxy for pre-test metrics; however, inconsistencies in question formatting limited longitudinal comparisons such as feelings about working with large animals before the camp. Consequently, the 2023 study design was modified to utilize strictly matched pre- and post-test instruments, enabling a more robust statistical analysis.

Programmatic content also shifted due to site-specific resources and clinician availability. While the 2022 curriculum utilized rigid learning objectives, real-time instructional pivoting suggested that a broader “programmatic objective” framework was more effective for guest clinicians than granular session-specific goals. Furthermore, the 2023 transition to the OSU Veterinary Medical Center allowed for an expanded clinical scope, exposing participants to specialized tertiary care that was inaccessible at the Ohio State Fair (OSF) location.

Furthermore, pedagogical structure played a clear role in student engagement. In 2023, two sessions were primarily observational rather than hands-on, which correlated with lower scores regarding career interest. This trend mirrors the 2022 results, where the dairy cattle session, the non-tactile station, received the only disagrees or strongly disagrees to learning in that session. These findings underscore the necessity of experiential “hands-on” learning in maintaining student interest in large-animal medicine [26].

Location can also play a role in participants’ perceptions of FSVM careers. The 2022 event took place in a location with pristine conditions and animals that were easy to work with, along with other simulations. Our 2023 year was on a working farm that offered a more realistic feel of FSVM work space. This, along with the difference in pedagogical structures, could result in a decline in participants wanting to enter a FSVM career.

The 2023 recruitment strategy was changed to address the low attendance of participants from urban areas. To address this, partnerships with local health departments, Boys and Girls Clubs, and the YMCA were highly effective in reaching the target urban demographic. However, logistical barriers remain, including the reliance on private transportation to rural research facilities, which could exacerbate pre-existing transportation insecurities [27]. Another logistical barrier is that scheduling sessions during standard work hours may inadvertently exclude low-socioeconomic status (SES) families who would not be able to take off the time to bring participants to the camp.

To improve the “pipeline” for urban students, future iterations should consider “bringing the farm to the city” by utilizing mobile clinics or small ruminant models in urban community centers. Partnering with established municipal summer programs and public transportation routes could further mitigate accessibility issues. By decentralizing the learning environment and ensuring consistent hands-on interaction, FSVM outreach can become more inclusive.

Several limitations warrant consideration when interpreting the outcomes of this study. First, the geographic and temporal scope was restricted to two iterations of a single program in Ohio, which may limit the generalizability of the findings to other regions with different agricultural landscapes or demographic profiles. Second, the sample size, particularly for the urban-targeted 2023 cohort, was relatively small; larger longitudinal studies are required to confirm whether these short-term attitudinal shifts persist through undergraduate education. Third, the use of meal vouchers as incentives for completing the post-test in the 2022 vet camp could have made filling out the survey more crucial to some participants based on socioeconomic status.

Additionally, a degree of selection bias is inherent in voluntary outreach programs, as students who choose to attend a veterinary camp likely possess a baseline interest in animal science. This complicates the ability to isolate the program’s impact from pre-existing career motivations. Another bias to be addressed is the social desirability bias. Post-tests were directly handed to staff members for collection. This could have intimidated some participants to not want to fill out the tests negatively. With our association with the veterinary college, participants may also want to give the camp favorable reviews in hopes of studying at the college. Furthermore, shifting from hands-on clinical tasks to observational demonstrations due to animal welfare and site-specific constraints introduced a variable that appeared to influence participant engagement and career intent. Finally, while the post-test data indicate an immediate increase in knowledge and interest, this study does not include long-term tracking data to verify participants’ eventual matriculation into veterinary medical programs. Future research should prioritize longitudinal tracking to evaluate the actual conversion rate of camp participants into the food supply veterinary workforce.

The “Be a Vet for a Day” program demonstrates that targeted, experiential outreach can effectively bridge the gap between urban youth and the food supply veterinary medicine (FSVM) sector. By evolving the recruitment strategy from general social media outreach to direct community partnerships, the program successfully shifted its demographic to include a majority of students from non-farm backgrounds. This shift is critical to diversifying the veterinary pipeline and addressing the ongoing workforce shortages in rural and production medicine.

Key findings indicate that while live-animal interaction remains the primary driver of student engagement and satisfaction, realistic exposure to the demands of herd health and biosecurity provides students with the necessary context to make informed career decisions. The significant increase in biosecurity knowledge (*p* = 0.002) suggests that even short-term interventions can achieve measurable educational gains.

Future initiatives should prioritize logistical accessibility, such as urban-based programming or providing transportation, to ensure that socioeconomic factors do not serve as a barrier to professional entry. Ultimately, programs like these at The Ohio State University provide a replicable model for veterinary colleges to engage the next generation of food supply veterinarians, ensuring the continued resilience of the global food system.

## Figures and Tables

**Table 1:** Demographics of registered participants in Be a Vet for a Day Camp Year 1 (*N* = 109).

Demographics of Participants	Mean	Max	Min
Age	16	21	13
Grade Leaving	Proportion		
9th	30.3%		
10th	26.6%		
11th	27.5%		
12th	12.8%		
College	2.8%		

**Table 2:** Year 1—2 × 2 This table compares reported interest in FSVM careers on registration (pre-survey) (*N* = 112) and after participation in Be a Vet for a Day (post-survey) (*N* = 69) in the 2022 camp. FSVM interest was assessed according to which field of veterinary medicine was indicated on the participant’s registration (pre-survey).

	FSVM Interest	Non-FSVM Interest	Total
Post-Survey	55	14	69
Registration (Pre-Survey)	67	45	112

**Table 3:** Demographics of Be a Vet for a Day Year One and Two comparisons shown in proportions. Includes age, gender, race, and residence demographics of those attended.

		Mean	Maximum	Minimum
Age	(Year 1)	16	19	13
	(Year 2)	16	18	14
			Proportion	
Gender			Year 1 (*N* = 69)	Year 2 (*N* = 39)
Male			11.6%	7.7%
Female			88.4%	87.2%
Non-Binary			-	5.1%
Race			*N* = 67	*N* = 38
American Indian/Alaskan Native, African American, White			-	2.6%
American Indian/Alaskan Native			3%	-
African American			1.5%	13.2%
White			95.5%	84.2%
Residence			*N* = 68	*N* = 40
Farm			60.3%	32.5%
Town (Under 10,000 population and rural non-farm)			16.2%	10%
Town/City (10,000–50,000 population and suburb)			11.8%	30%
Suburb of City (>50,000 population)			7.4%	22.5%
Central City (>50,000 population)			4.4%	5%

**Table 4:** Year 2—Participants pre- and post- surveys by percentage for the 2nd year of camp (2023), reported in proportions.

Questions	Strongly Agree/ Agree	Strongly Disagree/ Disagree
(1) Am aware of opportunities in food animal veterinary medicine.		
Pre (*N* = 39)	94.9%	5.1%
Post (*N* = 41)	100%	0%
(2) Am considering a career in food animal veterinary medicine.		
Pre (*N* = 40)	75%	25%
Post (*N* = 41)	73.2%	26.8%
(3) Plan to pursue a career in food animal veterinary medicine.		
Pre (*N* = 40)	80%	20%
Post (*N* = 40)	60%	40%
(4) Feel comfortable working with large animals such as horses and cattle.		
Pre (*N* = 40)	97.5%	2.5%
Post (*N* = 40)	97.5%	2.5%
(5) Understand the importance of large animal veterinary medicine.		
Pre (*N* = 39)	100%	0%
Post (*N* = 41)	100%	0%
(6) Know what biosecurity entails on a farm.		
Pre (*N* = 39)	74.4%	25.6%
Post (*N* = 41)	100%	0%

**Table 5:** Year 2—Results of McNemar’s test, wherein A, B, C, and D indicate a 2 × 2 table entered with A starting in the upper left corner and read left to right. Post-tests were placed on the rows and pre-tests were placed on the columns. A represents strongly disagree/disagree for both the pre- and post-test and D represents strongly agree/agree in the pre- and post-test. Incomplete pre- and post-tests were not utilized in this data set (n = 36). Significant values are italicized in the specified column. Question numbers correspond with Table 4 questions (*p* > 0.05).

Question	A	B	C	D	Exact	*p*-Value
1	0	2	0	34	0.50	0.157
2	5	4	6	21	0.7539	0.527
3	7	1	8	20	0.0391	0.0196
4	1	0	0	35	1.00	-
5	0	0	0	36	1.00	-
6	0	10	0	26	0.0020	0.0016

## Data Availability

The original contributions presented in this study are included in the article. Further inquiries can be directed to the corresponding author.

## Abbreviations

The following abbreviations are used in this manuscript:

FSVM	Food Supply Veterinary Medicine
VCPR	Veterinary Client Patient Relationship
AMR	Antimicrobial Resistance
STEM	Science, Technology, Engineering and Math
CFAES	College of Food Agriculture and Environmental Sciences
OSU	Ohio State University
OVMA	Ohio Veterinary Medical Association
OSF	Ohio State Fair
CVM	College of Veterinary Medicine
PPE	Personal Protective Equipment
